# Diagnostic performance of abdominal point of care ultrasound performed by an emergency physician in acute right iliac fossa pain

**DOI:** 10.1186/s13089-018-0112-5

**Published:** 2018-11-23

**Authors:** Jean-Eudes Bourcier, Emeric Gallard, Jean-Philippe Redonnet, Magali Majourau, Dominique Deshaie, Jean-Marie Bourgeois, Didier Garnier, Thomas Geeraerts

**Affiliations:** 1 Emergency, Anesthesiology and Critical Care Department, Lourdes Hospital, 2 Avenue Alexandre Marqui, 65100 Lourdes, France; 2 CFFE (Centre Francophone de Formation en Echographie), Nîmes, France; 3 Anesthesiology and Critical Care Department, Toulouse University Hospital, University Toulouse III Paul Sabatier, Toulouse, France

**Keywords:** Bedside ultrasound, Abdominal pain, Appendicitis, Diagnostic methods

## Abstract

**Background:**

Right iliac fossa abdominal pain is a common reason for emergency ward admissions, its etiology is difficult to diagnose. It can be facilitated by an imaging examination, such as a Computerized Tomography scan which exposes the patient to ionizing radiation and implies delays. A bedside ultrasound performed by emergency physicians could avoid these issues. The aim of our study was to assess the performance of ultrasound carried out at the patient’s bedside by an emergency physician compared with a clinical-laboratory examination for the diagnosis of a surgical pathology in right iliac fossa pain.

**Methods:**

This is a single-center prospective cohort study conducted in an Emergency Department receiving 19,000 patients per year. All patients presenting pain in the right iliac fossa were included by four (out of ten) emergency physicians certified in an ultrasound examination. A full grid pattern scan ultrasound of the abdominal cavity with analysis of the right iliac fossa was performed. The primary outcome was to compare the diagnosis performance of bedside ultrasound and clinical-laboratory examination to detect a surgical pathology. Two emergency physicians who did not participate in the study made the final diagnosis (i.e., surgical or non-surgical pathology) by reviewing the entire medical chart of each patient.

**Results:**

From January 2011 to July 2013, 158 patients with a median age of 17 [13–32] years were analyzed. The diagnosed cases were: appendicitis (53), non-specific abdominal pain (48), lymphadenitis (22), ileitis (11), complicated ovarian cysts (7), neoplasias (5), inflammatory or infectious colitis (5), inguinal herniations (3), bowel obstructions (2), and salpingitis (2). The accuracy of ultrasound diagnoses was 0.89 (95% CI 0.84–0.94) versus 0.70 (95% CI 0.57–0.82) for diagnoses based on clinical-laboratory examination only (*p* < 0.001).

**Conclusion:**

Bedsides, ultrasound allows an accurate diagnosis of a surgical pathology in 89% of cases, which is more efficient than the clinical-laboratory examination.

## Introduction

Abdominal pain is the third most common reason for attending a hospital emergency ward [1]. Even though in 40% of cases the clinical picture is benign and without a precise etiology; in cases requiring emergency surgery, any delay in treatment can result in an increase in morbidity or mortality. The pain in the right iliac fossa represents a specific problem in regard to appendicitis, as the diagnosis is sometimes hard to make, with a clinical exam and a laboratory test alone [2, 3]. On their own, clinical examinations perform relatively poorly, with sensitivity and specificity ranging from 50 to 86% [4], thus warranting introduction of decisions scores. For instance, the Alvarado or Appendicitis Inflammatory Response (AIR) score, that couple clinical and laboratory assessments have sensitivity and specificity close to 90% according to some papers [5–9]. All the international guidelines are in support of the use of complementary examinations, and particularly of imaging, for undertaking a diagnosis of pain in the right iliac fossa [10–12]. The abdominal computerized tomography (CT) scan with injection of a contrasting agent is the standard imaging method, although it has drawbacks with regard to availability and the associated radiation [13, 14]. Recently, imaging by magnetic resonance has become more common, particularly with children and pregnant women, although its availability is even more limited than for the CT scan [15–17]. Abdominal ultrasound has the advantage of being inexpensive and it does not involve ionizing radiation, although its performance remains operator dependent [18]. In practice, however, a radiologist cannot always be available on short notice, which can lead to diagnostic delays that may have detrimental consequences for the patient [19]. That is why an abdominal ultrasound performed by the emergency physician at the patient’s bedside in addition to clinical and biological data could be helpful in detection of a surgical pathology more efficiently. Of course, it remains dependent on the extent of training and the reliability of the interpretation of the imaging results by a non-specialist in radiology. Some studies have showed the reliable accuracy in the diagnosis of acute appendicitis, with a bedside ultrasound performed in emergency ward [20, 21].

The aim of our study was to assess the performance of abdominal ultrasound carried out at the patient’s bedside by an emergency physician compared with a clinical-laboratory examination, for the diagnosis of a surgical pathology in right iliac fossa. Our hypothesis was that ultrasound would improve diagnostic performance.

## Materials and methods

### Study design

This prospective cohort study was carried out in the emergency ward of a general hospital center that has 19,000 admissions per year.

### Ethics approval

The protocol for the study was approved by the Ethics Committee of Lourdes hospital (PV N°14244) and follows the World Medical Association’s Declaration of Helsinki. It did not require written consent since the standard treatment procedures were not altered in order to perform the study.

### Patients

From January 2011 to July 2013, all patients (children and adults) presented to the emergency department with as main symptom an acute pain of the right iliac fossa were included if a physician trained in use of ultrasound was present.

### Clinical and laboratory examination

The history of the patient and the clinical examination strived to characterize the surgical origin of the pain by relying on standard functional and physical symptoms (e.g., signs of occlusion or localized peritonitis, nausea, vomiting, a palpable mass, abdominal pain, rebound tenderness, etc.). Urological and/or gynecological symptoms were also probed for, depending on the symptoms. A point of care urinalysis was also performed at the admission.

Systematic common laboratory testing upon admission was composed of a blood count, a test for C-reactive protein levels, as well as a blood electrolytes test, and a test of kidney function. Depending on the patient history and clinical findings, a Human Chorionic Gonadotropin (β-HCG) test, a liver and pancreatic assessment, a hemostasis assessment, a blood group typing, or any other laboratory test deemed to be of use by the emergency physician were added.

Upon assessment of all these items, the physician had to make a suspected clinical-laboratory diagnosis: surgical pathology (i.e., requiring a surgical treatment) or medical pathology (i.e., requiring a medical treatment).

### Ultrasound data

After having undergone basic training in the use of abdominal ultrasound imaging, the emergency physicians who included patients for this study had to have at least 2 years of experience with emergency ultrasound use. This was first broken down into training sessions in a certified center over 7 days and then by e-learning for 1 year. This training was based on the specifications of the American College of Emergency Physicians [22]. This course was completed by supervised training over a 6-month period that involved receiving ultrasound images with validation of the observed anomalies made by a radiologist.

Following the clinical examination and the laboratory testing, the emergency physician performed an ultrasound at the patients’ bedside (using an M Turbo miniaturized ultrasound device from FUJIFILM SONOSITE©, Bothell, WA, USA).

The examination consisted of an initial grid pattern of the abdominal cavity using a convex abdominal probe (3.5 MHz), which permitted to search peritoneal effusion, masses. A scan of the two kidneys, liver, biliary tracts and aorta was equally performed. Because of the lack of a vaginal probe, pelvic analysis was performed by an abdominal probe. The emergency physician used the surface probe (7.5 MHz) to complete the scan, guided by the site of the pain, the analysis then focused on the right iliac fossa. Gradual compression was applied as described by Ooms [23, 24]: the normal digestive structures, which contain air, disappear from the screen upon compression. Furthermore, their wall is thin, with a thickness of less than 3 mm. By contrast, an inflamed digestive tract is not compressible, and it is painful upon application of pressure, while also exhibiting a thickened wall.

**Fig. 1:**
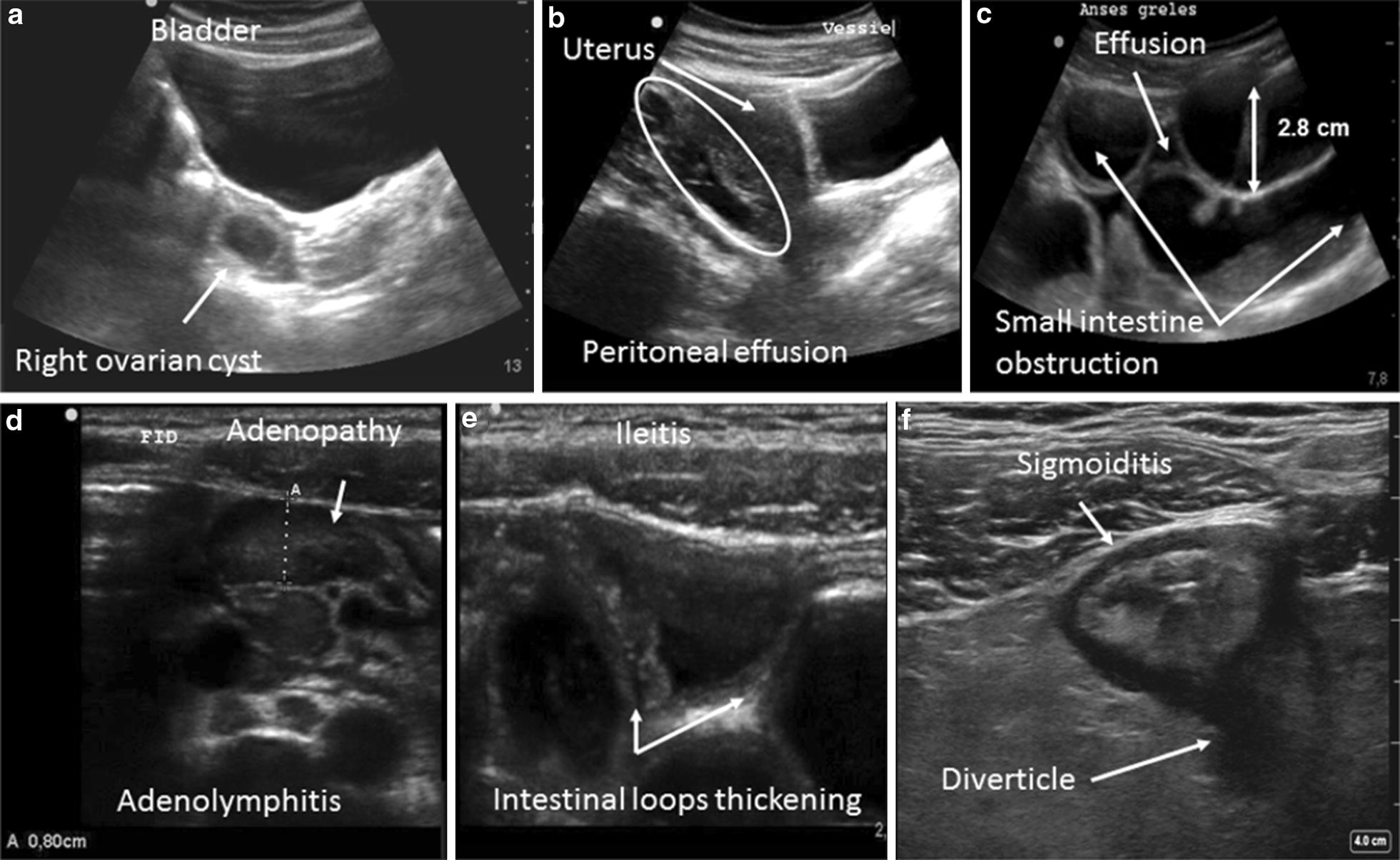
Examples of abnormal ultrasound images: **a** a latero-uterine mass with a sonolucent liquid content suggestive of a complicated ovarian cyst, **b** isolated peritoneal effusion (often at the bottom of the Douglas pouch), **c** appearance of a small intestinal occlusion (widening of the intestinal loops by more than 2.5 cm, with a liquid content and possible inter-loop effusion), **d** adenopathies (hypoechoic oval images greater than 5 mm in terms of the antero-posterior diameter, sensitive to passage of the probe, and enhanced by color Doppler), **e** thickening of the last small intestinal loops suggestive of ileitis (thickening by more than 3 mm of the wall of the digestive tract), **f** circumferential widening of the colon wall (a pseudo-kidney appearance) suggestive of an inflammation or an infection

Other pathological images can also be seen, such as an intra-peritoneal effusion, an obstruction syndrome, inflammatory adenopathies, and complicated ovarian cyst [25–27] (Fig. 1).

**Fig. 2:**
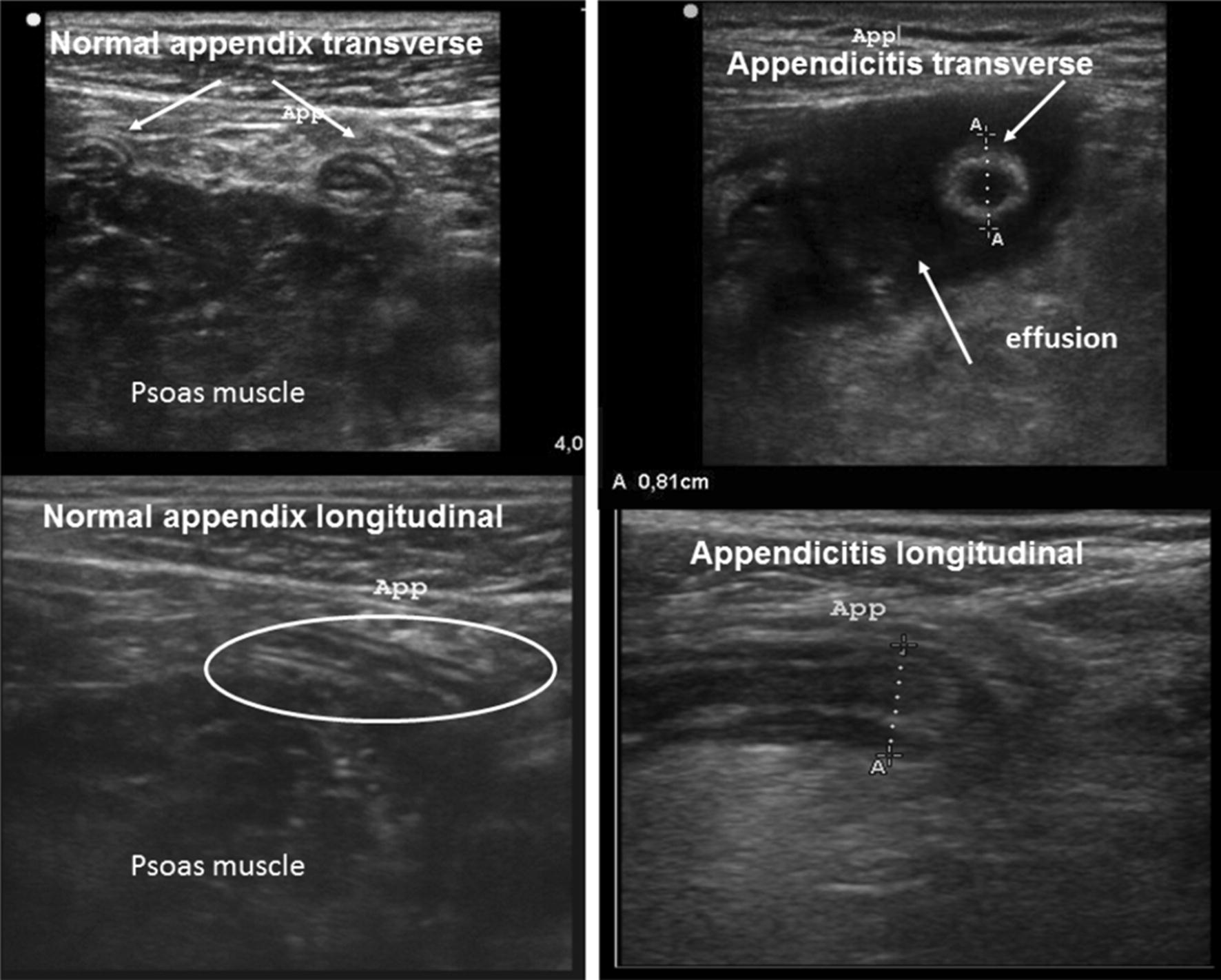
Ultrasound appearance of a normal appendix and a pathological one

In all cases, the emergency physician attempted to adequately discern the appendix. A diagnosis of appendicitis was made in conjunction with more than two of the following images: a double cockade cross-sectional appearance of the appendix of more than 6 mm in diameter, ending as a sleeve in a longitudinal section, a non-compressible nature, and association or not of a peri-appendicular effusion. In case of normal appendix viewed (none of previous images), this diagnosis was excluded (Fig. 2).

Upon completion of the examination, the practitioner provided a full written report regarding the ultrasound that fully detailed all of their findings. In case of a visually diseased appendix, intestinal occlusion, or peritoneal effusion, the ultrasound indicated a surgical pathology. In case of a normal appearing appendix, or other images indicative of pathologies, the ultrasound indicated a medical pathology.

After making the clinical-laboratory diagnosis, and the ultrasound diagnosis, the emergency physician in charge of the patient could ask for an abdominal CT scan and/or the opinion of a surgeon if he deemed it necessary, in particular to decide on the continuation of the patient’s care.

### Final diagnosis

The final diagnosis was the etiologic diagnosis of right iliac fossa pain (i.e., surgical or medical pathology). A surgical pathology was defined by a pathology requiring surgical treatment like laparoscopy or laparotomy which was decided and performed by the surgeon. Medical pathology was defined by a pathology requiring medical treatment (non-surgical).

Two emergency physicians who did not participate in the study made the final diagnosis by reviewing the entire medical chart of each patient, taking into account their evolution. They had to classify patients into two groups: surgical or medical pathology. In each group, the etiologic diagnosis was investigated.

For the patients who underwent surgery, the final diagnosis relied on an analysis of the surgical report and of the histological examination in case of surgical exeresis. For the patients who were hospitalized without undergoing surgery, the final diagnosis was based on the final hospitalization report. When patients were not hospitalized, they were contacted 2 months after discharge, and the diagnosis made when they left the emergency ward was then confirmed or not by their medical progress. Indeed, a surgical pathology was excluded if there was no re-hospitalization in our institution or another. In case of discrepancy between the two emergency physicians, the opinion of a surgeon from our institution could be taken into consideration.

Consequently, the performance of abdominal ultrasound was compared to the performance of clinical-laboratory examination for the diagnosis of a surgical pathology.

### Statistical analyses

The quantitative variables were expressed as mean ± SD for normally distributed variables and otherwise as a median with interquartile range. Qualitative variables were expressed as numerical values and as percentages.

The diagnostic performances of the ultrasound and the clinical and laboratory examination were expressed as sensitivity (Se), specificity (Sp), positive predictive value (PPV), negative predictive value (NPV) with their [confidence intervals at 95%]. A Youden’s index and an accuracy were calculated. The Youden’s index is defined by (sensitivity + specificity − 1), and the performance of the examination was deemed to be better when the Youden’s index is close to 1. Accuracy was defined as the percentage of properly classified cases upon completion of examinations for which the performance was tested.

The relative accuracies of the ultrasound and of the clinical-laboratory diagnosis were compared using a McNemar’s test, with a threshold for significance set at *p* = 0.05.

No power analysis was performed because of the absence of previous studies comparing abdominal ultrasound by emergency physician and standard examination (clinic + laboratory) in the management of right iliac fossa pain.

## Results

Four emergency physicians (of ten in the department) met the training requirements and thus participated in the study.

Ultimately, 158 patients were analyzed (sex ratio = 1) who were 17 [13–32] years old. Of these, 69 patients (44%) were hospitalized and 89 (56%) were sent home. The time spent in the emergency ward was 167 ± 51 min. The mean Body Mass Index was 21 ± 4 kg m^−2^.

The final diagnoses made included 53 cases of appendicitis (33%), 48 (31%) cases of non-specific abdominal pain (NSAP), 22 cases of adenolymphitis (14%), 11 cases of ileitis(7%), 7 complicated ovarian cysts (5%), 5 (3%) newly discovered neoplasias (4 colon, 1 pancreatic), 5 (3%) cases of inflammatory or infectious colitis (3 sigmoidal, 2 inflammatory colitis), 3 (2%) inguinal herniation’s, 2 (1%) bowel obstructions (one by a bridle and one by a neoplastic growth) and 2 (1%) salpingitis.

**Fig. 3:**
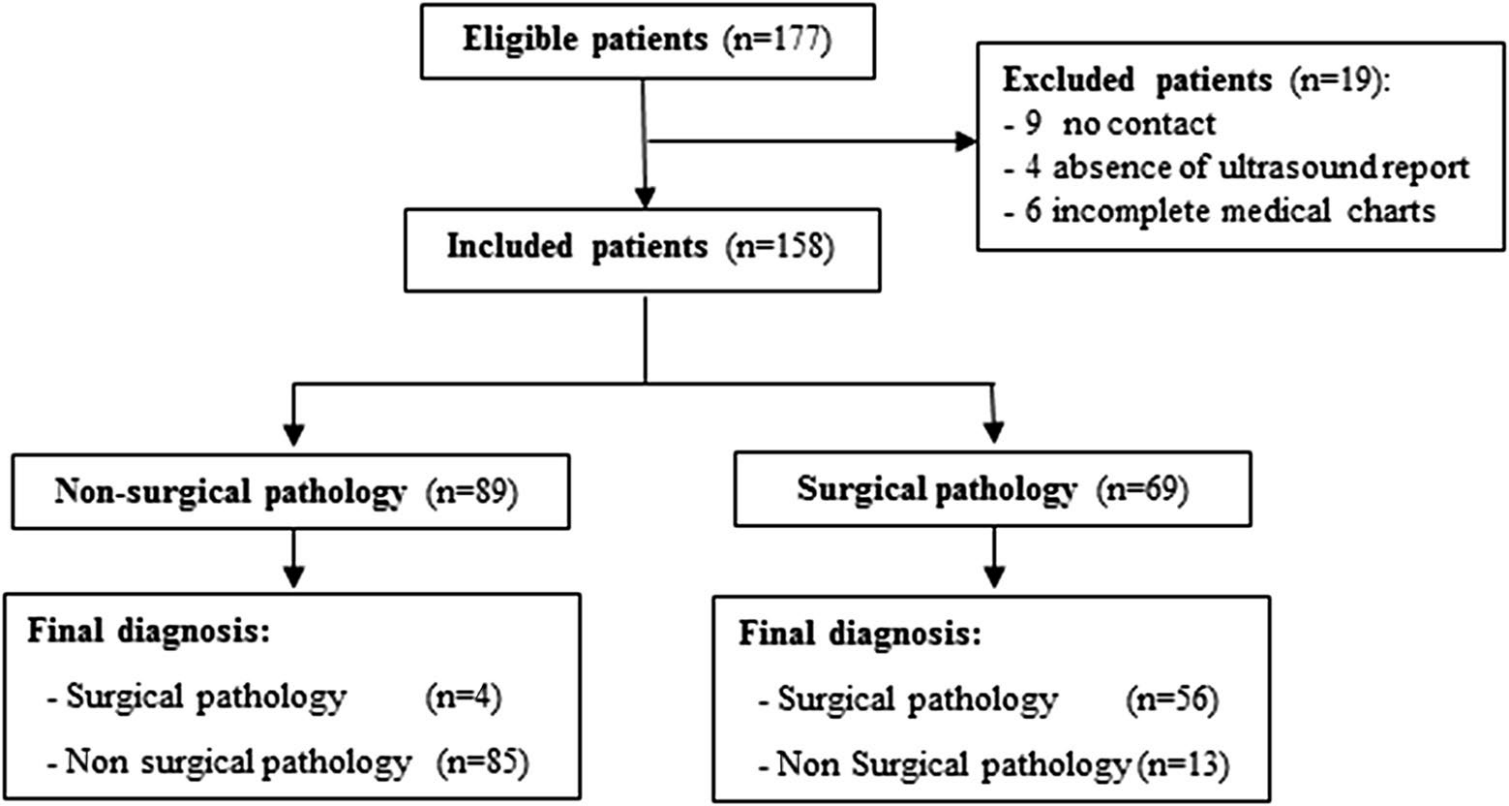
STARD flow diagram

The sample characteristic is presented in STARD flow diagram (Fig. 3).

Detailed images viewed on ultrasound compared to the final diagnosis are reported in Table 1.

**Table 1:**
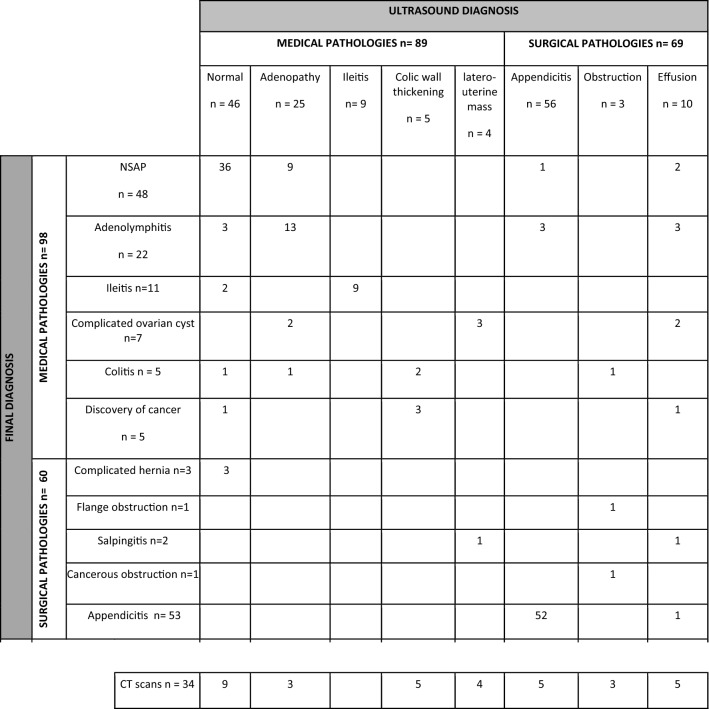
Comparison of ultrasound diagnosis and final diagnosis

Out of the 56 patients with an appendicitis diagnosed by ultrasound, 44 (84%) were taken directly to the operating room after surgical advice in the emergency department. There were four false-positive ultrasounds where the final diagnosis turned out to be one NSAP and three adenolymphitis.

None of these four patients underwent an operation, because they were examined by the surgeon on duty. This one decided to supervise three patients in the surgery department. Concerning the fourth he asked for a CT scan.

Among the 89 patients for whom the bedside ultrasound did not find a surgical pathology, there were four false negative. The diagnosis was corrected by surgeon on duty for two of them and by a CT scan for two others. For the other eighty-five patients (true negative), none had to later undergo an operation or be re-hospitalized.

The clinical-laboratory diagnosis compared to the final diagnosis is shown in Table 2.

**Table 2:**
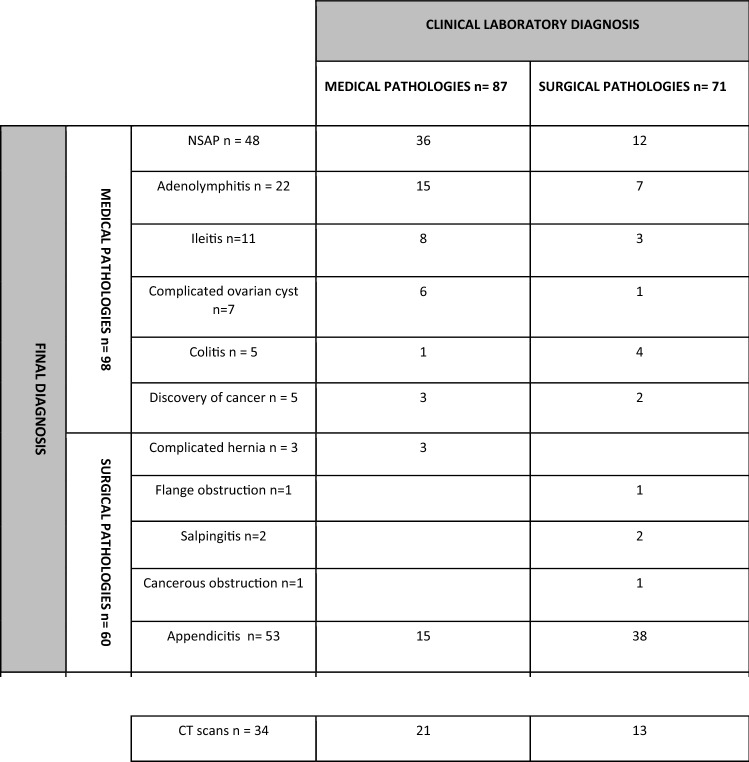
Comparison of the clinical-laboratory diagnosis and the final diagnosis

The compared performances of bedside ultrasound and clinical-laboratory approach according to the diagnosis of a surgical pathology are reported in Table 3.

**Table 3:** Performance of ultrasound and clinical-laboratory examination according to the final diagnosis of surgical pathology

	Sensitivity [95% CI]	Specificity [95% CI]	PPV [95% CI]	NPV [95% CI]	Youden	Accuracy * [95% CI]
Ultrasounds	0.93 [0.87, 0.99]	0.87 [0.79, 0.93]	0.81 [0.72, 0.90]	0.95 [0.91, 0.99]	0.80	0.89 [0.84, 0.94]
Clinical-laboratory examination	0.7 [0.58, 0.81]	0.7 [0.60, 0.78]	0.59 [0.46, 0.69]	0.79 [0.7, 0.87]	0.4	0.70 [0.57, 0.82]

95% CI: confidence interval; PPV: positive predictive value; NPV: negative predictive value

* *p* < 0.05

## Discussion

In our study of patients exhibiting pain in the right iliac fossa, an ultrasound performed by the emergency physician allowed for the diagnosis of a surgical pathology with an accuracy rate of 89%. This amounts to a significant gain in performance relative to clinical-laboratory examinations, since the latter has an accuracy rate of 70%.

The performance of the clinical-laboratory approach is relatively modest, perhaps because there is still ample room for improvement in terms of the clinical examination. This is indeed suggested by studies of this approach, and Alvarado or AIR score also yields extremely variable results [28–31]. These scores are used to determine likelihood of appendicitis. However, this affection is not the only one that can underlie pain located in the right iliac fossa, thus explaining the lower performance with a cohort of right iliac fossa pain comprising different pathologies. When the final diagnosis was appendicitis, only 38 out of 53 cases were suspected by the clinical-laboratory examination, which seem to confirm that these scores are not foolproof. Analysis of the results shows that the highest levels of false positives were encountered above all in cases of adenolymphitis and ileitis, or in cases of colitis (inflammatory or infectious). This can undoubtedly be explained by the early appearance of these affections with surgical pathology such as appendicitis.

As for the 13 patients for whom the ultrasound diagnosis indicated a surgical pathology, although the final diagnosis was in fact a non-surgical pathology, none incorrectly underwent surgery. This was thanks to reassessment by the surgeon on duty, who either monitored them in the surgery department, or who ordered a CT scan that allowed the diagnosis to be corrected or to be made with a greater degree of accuracy. In case of discordant ultrasound/clinical–biological results, the reassessment of the surgeon evaluation was considered as the reference method.

The performances of the ultrasound carried out by the emergency physician are comparable to those found in the literature when a radiologist performed the ultrasound: i.e., a specificity of 70 to 90% and a sensitivity of 85 to 100%. These works compared accuracy of an ultrasound with the surgery results or a CT scan only in appendicitis diagnosis [24, 32]. By contrast, assessments regarding the performance of ultrasounds by doctors who are not radiologists encountered highly variable results (sensitivity from 60 to 90%) [33–35]. This was probably related to the fact that the duration of learning to research appendicitis was too short; however, the emergency ultrasound guidelines do not yet cover this area [22].

The fact that the emergency physician performs the ultrasound themselves will strengthen the notion of the pain in the right iliac fossa being innocuous when they do not see an abnormal image and, by contrast, tend to confirm or give rise to suspicion of a serious diagnosis when they obtain an abnormal image. This is the case for appendicitis, unlike with other pathologies, where a typical image usually is enough to confirm a diagnosis. Indeed, colonic afflictions (inflammatory, infectious, or neoplastic) translate into a non-specific thickening of the wall. Similarly, a peritoneal effusion, even when in isolation, is reason for the practitioner to be cautious since it should be considered as a sign of a surgical pathology until proven otherwise. These elements are reason to prescribe complementary assessments or to seek specialist advice.

Thus, this strategy allowed scanning to be optimized, with the irradiative examination given to 28% of the patients in our study, whereas in North American register, a CT scan was performed for 43% of patients who suffered from abdominal pain [36, 37].

Furthermore, this ultrasound-clinical process optimizes the processing of the patient by avoiding incorrect conclusions being made with regard to the cause of harmless abdominal pain, and by unnecessarily resulting in invasive procedures or hospitalization. As such, none of the 86 patients who were processed by the emergency department secondarily presented surgical pathologies.

With regard to the 60 patients in our cohort who underwent a surgical treatment, the use of an ultrasound resulted in rectifying the diagnosis for 14 (23%) patients for which the clinical-laboratory approach concluded with a non-surgical pathology.

These findings could encourage a two-step approach, with first clinical-laboratory and bedside ultrasound examination and secondarily a CT scan and/or surgical advice for management of right iliac fossa pain [38].

## Limitations

As this is a monocentric study with a relatively limited number of participants, it is difficult to extrapolate the results of this work to the entire specialty.

Our results are probably not transferable to less well-trained operators. Nonetheless, the results of this work are reason to encourage further training efforts.

In the present sample of patients, no pyelonephritis or ureteral colic was observed. This could be related to strict inclusion criteria (main symptom of acute pain of the right iliac fossa). The number of patients included in the study did not reflect the prevalence of right iliac fossa pain in the emergency department over 30 months because only 4 of 10 EP could participate in the study.

Despite a good performance by the ultrasound-clinical strategy, there were still 17 diagnostic errors (thirteen false positives and four false negatives).

Three of the four surgical pathologies not detected by the ultrasound were strangulated inguinal hernia, probably explained by the fact that the ultrasound scan was not carried out in this area. It should be noted that clinical examinations for this affliction were equally inadequate. This highlights the importance of systematically exploring this area, with a careful physical examination and a focus with an ultrasound probe.

The 13 false positives were largely dominated by the presence of a non-specific peritoneal effusion in eight (61%) cases. Indeed, the cohort included only two cases out of 10 where those in fact revealed a surgical pathology. This is why it seems reasonable to retain this warning sign, since it motivates further exploration. Regarding the four false positives for appendicitis, it was the reassessment by the surgeon that allowed a cutoff decision to be made. The latter hence has a central role in the assessment of acute abdominal pains [39, 40].

Our analysis of the performance of the ultrasound was based on whether an anomaly was seen rather than whether an accurate diagnosis was made. This is particularly true in case of peritoneal effusion which can be indirect sign of a surgical pathology. This choice potentially leads to an overestimation of the merit of the ultrasound performed by the emergency physician.

As this study was not comparative, it does not allow for the contribution of the ultrasound to the diagnostic performance of the emergency physician to be appraised. For that it would have been necessary to compare the treatment with a typical process whereby the examinations were performed by a radiologist.

Similarly, it was not possible to assess the gain in terms of the effect on the duration of the stay in the emergency department.

The emergency physicians were aware of clinical-laboratory results when ultrasounds were performed. This could have influenced the results. Indeed, confirmation of the final diagnosis by the two independent doctors was performed retrospectively, as was the case for the complementary examinations, of which the bedside ultrasound could influence the diagnosis. Yet this is so for all the complementary examinations (laboratory tests and CT scans) which form an integral part of the diagnostic process. Furthermore, this limitation does not apply to the patients who underwent surgery. Aside from surgical, anatomical–pathological, or scan-based findings, confirmation of the final diagnosis was not supported by items that could not be questioned. Indeed, it is ethically questionable to perform a CT for all patients or to make surgical exploration like laparoscopy in order to exclude a surgical pathology in case of adenolymphitis, ileitis or NSAP for example. Yet this limitation can be found in most of the studies treating abdominal pains, and particularly in the case of a non-surgical diagnosis [41, 42].

## Conclusion

The diagnosis accuracy of an abdominal ultrasound performed by an emergency physician is 89% compared to an accuracy of 70% by a clinical-laboratory examination alone, for the diagnosis of a surgical pathology in case of right iliac fossa pain.

## Abbreviations

CT: Computerized Tomography
PPV: positive predictive value
NPV: negative predictive value
